# Unravelling the genetic variability of host resilience to endo- and ectoparasites in Nellore commercial herds

**DOI:** 10.1186/s12711-023-00844-9

**Published:** 2023-11-21

**Authors:** Gabriela Canabrava Gouveia, Virgínia Mara Pereira Ribeiro, Marina Rufino Salinas Fortes, Fernanda Santos Silva Raidan, Antonio Reverter, Laercio Ribeiro Porto-Neto, Mariana Mamedes de Moraes, Daniel Resende Gonçalves, Marcos Vinicius Gualberto Barbosa da Silva, Fabio Luiz Buranelo Toral

**Affiliations:** 1 Departamento de Zootecnia, Escola de Veterinária, Universidade Federal de Minas Gerais, Belo Horizonte, Brazil; 2 School of Chemistry and Molecular Bioscience, The University of Queensland, Brisbane, QLD Australia; 3 Queensland Alliance for Agriculture and Food Innovation, The University of Queensland, Brisbane, QLD Australia; 4 Agriculture and Food, Commonwealth Scientific and Industrial Research Organization (CSIRO), Brisbane, QLD Australia; 5 Swine Business Unit, Hendrix Genetics, 5831 CK Boxmeer, The Netherlands; 6 Mundo Novo Farm, Uberaba, Brazil; 7 Embrapa Gado de Leite, Empresa Brasileira de Pesquisa Agropecuária, Juiz de Fora, Brazil

## Abstract

**Background:**

Host resilience (HR) to parasites can affect the performance of animals. Therefore, the aim of this study was to present a detailed investigation of the genetic mechanisms of HR to ticks (TICK), gastrointestinal nematodes (GIN), and *Eimeria* spp. (EIM) in Nellore cattle that were raised under natural infestation and a prophylactic parasite control strategy. In our study, HR was defined as the slope coefficient of body weight (BW) when TICK, GIN, and EIM burdens were used as environmental gradients in random regression models. In total, 1712 animals were evaluated at five measurement events (ME) at an average age of 331, 385, 443, 498, and 555 days, which generated 7307 body weight (BW) records. Of the 1712 animals, 1075 genotyped animals were used in genome-wide association studies to identify genomic regions associated with HR.

**Results:**

Posterior means of the heritability estimates for BW ranged from 0.09 to 0.54 across parasites and ME. The single nucleotide polymorphism (SNP)-derived heritability for BW at each ME ranged from a low (0.09 at ME.331) to a moderate value (0.23 at ME.555). Those estimates show that genetic progress can be achieved for BW through selection. Both genetic and genomic associations between BW and HR to TICK, GIN, and EIM confirmed that parasite infestation impacted the performance of animals. Selection for BW under an environment with a controlled parasite burden is an alternative to improve both, BW and HR. There was no impact of age of measurement on the estimates of genetic variance for HR. Five quantitative trait loci (QTL) were associated with HR to EIM but none with HR to TICK and to GIN. These QTL contain genes that were previously shown to be associated with the production of antibody modulators and chemokines that are released in the intestinal epithelium.

**Conclusions:**

Selection for BW under natural infestation and controlled parasite burden, via prophylactic parasite control, contributes to the identification of animals that are resilient to nematodes and *Eimeria* ssp. Although we verified that sufficient genetic variation existed for HR, we did not find any genes associated with mechanisms that could justify the expression of HR to TICK and GIN.

**Supplementary Information:**

The online version contains supplementary material available at 10.1186/s12711-023-00844-9.

## Background

Ecto- and endoparasites such as ticks (TICK), gastrointestinal nematodes (GIN), and *Eimeria* spp. (EIM) are endemic in tropical countries and responsible for several economic and productivity losses in cattle production systems [1]. Parasitic loads represent an important challenge for cattle production, especially in tropical countries, such as Brazil. An animal’s ability to respond to parasite loads is one of the stress factors that can impact the sustainability of the production system.

In the literature, different phenotypes are used to describe response to disease, among which we would like to highlight resistance, tolerance and resilience [2]. Resistance is the ability of the host to fully resist to infection, i.e., the ability to prevent parasite infection [3]. Tolerance is often described as the changes in the host’s fitness, once it is infected, with respect to the evolution of the internal pathogen burden [3]. In contrast, resilience (HR) can be measured as the ability of the host, once infected, to maintain its fitness regardless of the internal pathogen burden [2, 4–6]. As highlighted by Knap and Doeschl-Wilson [2], HR can be defined as a combination of both resistance and tolerance mechanisms. In summary, the main difference between the last two definitions is whether the levels of internal pathogen burden are considered (tolerance) or not (resilience).

HR can be estimated as a continuous trait using reaction norm models of the host’s performance on environmental stress factors [2]. Under the assumptions of the reaction norm model, different patterns of growth depending on parasite burden can be described. There is no study in the literature that has attempted to estimate the different relationships between these factors, and we have no strong indication of the most adequate assumption for this. Thus, assuming a simplistic linear relationship between growth and parasite burdens, the additive variance of a performance trait can be decomposed into three components: the intercept variance (i.e. the additive component of the variability in performance assuming the absence of stress factors), the slope variance (i.e. the HR), and the covariance between intercept and slope [7]. Therefore, when linear regressions are used, the genetic correlation between the intercept and slope coefficients quantifies the genetic association between performance and HR [8].

Reaction norm models have been used to estimate HR to *Fasciola hepatica* in Irish cattle [9] and resilience of Rainbow trouts to freshwater × seawater [10]. Furthermore, Mulder [11] showed that these models can be used in selection programs to improve response to selection for resilience. In our study, we estimated HR to TICK, GIN, and EIM using the host’s body weight and parasite burdens in random regression models. Therefore, our aim was to estimate genetic parameters for both BW and HR to TICK, GIN, and EIM and to identify genomic regions associated with these phenotypes in Nellore cattle.

## Methods

### Data collection and edition

We used the data on Nellore bulls that were born between 2010 and 2016 and raised on the Mundo Novo commercial farm, which is located in Uberaba, Minas Gerais state, Brazil (19° 24′ 33″ S and 48° 06′ 34″ W, at an altitude of 840 m, with a Monsoon-influenced humid subtropical climate or Cwa weather according to the Köppen scale). The Ethics and Animal Experimentation Committee of the Universidade Federal de Minas Gerais approved the experiment and data collection (Protocol 255/2010). Detailed description about the farm and the herd are in Passafaro et al. [12].

**Fig. 1:**
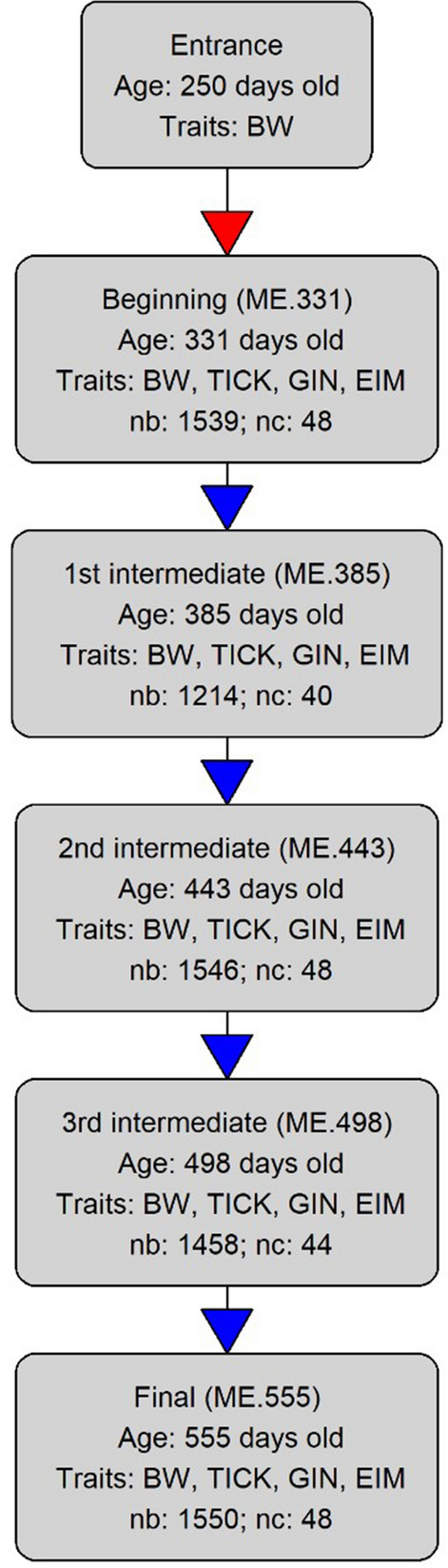
Diagram explaining data collection for performance tests of pasture raised cattle on the Mundo Novo farm—Brazil. Body weight (BW), ticks (TICK), eggs of gastrointestinal nematodes (GIN) and oocysts of *Eimeria* spp. (EIM) counts were collected at each measurement event (ME). “Age” represents the average age of animals at each ME. “nb” is the number of bulls and “nc” is the number of cohorts evaluated at each ME. Red arrow indicates a 70-day interval between evaluations, while blue arrows indicate a 56-day interval

The bulls were raised on pasture, which comprised mainly (> 80%) grass of the Uruchloa genus, with a stocking rate of approximately 0.98 animal unit per hectare. Animals had free access to mineral supplementation and clean water throughout the year. After weaning (210 days old on average), the males were evaluated in performance tests that lasted 294 days, and included 70 days of adaptation, to minimize potential nutritional and social stress, and 224 days of evaluation (Fig. 1). Animals that were evaluated together, in the same performance test, were raised under the same environmental conditions, ate grass of the same quality, and were subjected to similar social, adaptive, and environmental challenges for at least the last 56 days before the measurement events (ME).

The bulls were weighed at six ME: at day 1 of the performance test (data not used in our study), at the end of the adaptation period (day 70) and at four intervals of 56 days until the end of the test (Fig. 1), which defined five ME. The average age of the animals was 331, 385, 443, 498, or 555 days from the first to the fifth ME, respectively.

The tick counts used in the present study were obtained at each ME by counting the engorged female ticks, with a length size > 4.5 mm, on the right side of each animal [13]. The egg counts of gastrointestinal nematodes (GIN) and the oocyst counts of *Eimeria* spp. (EIM) were estimated by the number of eggs or oocysts per g of faeces, according to the modified McMaster technique [14].

Faecal samples were collected directly from the animals’ rectum using properly identified and lubricated plastic bags. They were then cooled and transferred into chilled coolers in the laboratory. To perform the counts, we diluted 2 g of faeces with 28 mL of water, prepared 2-mL aliquots of this mixture and mixed each aliquot with 2 mL of saturated Sheater’s solution (500 g of sugar, 6.5 mL of phenol and 360 mL of water). Then, a McMaster chamber was filled with 0.15 mL of the final solution to perform the counts of eggs and oocysts. Thus, in this study, tick, egg, and oocyst counts are the real counts observed on the right side of each animal or in the McMaster chamber.

Cohorts were defined by the combination of contemporary group (i.e. animals evaluated at a same performance test) and ME and only cohorts with more than five individuals were considered, thus, 7307 body weight records and parasite counts on 1712 animals were evaluated in this study (see Additional file 1: Table S1 and Additional file 2: Fig. S1). The pedigree file included 5944 animals from approximately nine generations, with 130 sires (with an average of 13.17 ± 12.46 offspring) and 1132 cows (with an average of 1.51 ± 0.77 offspring). The number of generations in the pedigree (generation coefficient) was calculated as follows:

$${GC}_{i}=\left(\frac{{GCS}_{i}+{GCD}_{i}}{2}\right)+1,$$

where ${GC}_{i}$ is the generation coefficient of the individual $i$; ${GCS}_{i}$ is the generation coefficient of the sire of animal $i$; and ${GCD}_{i}$ is the generation coefficient of the dam of animal $i$ [15]. Individuals with no known parent have a $GC$ equal to one, which means that they belong to the base population. In the present study, the population had an average generation coefficient ± standard deviation of 5.55 ± 2.45, ranging from 1 to 9.81. The summary statistics for the data used in the present study are presented at Table 1.

**Table 1:** Summary statistics for age at weighing (age), body weight (BW), tick (TICK), gastrointestinal nematode (GIN), and *Eimeria* spp. (EIM) counts at five measurement events (ME) in Nellore bulls

Trait	n	Mean	sd	Median	Min	Max
ME.331						
Age (days)	1539	330.72	23.49	334	275	373
BW (kg)	1539	223.08	33.23	220	138	343
TICK	1539	5.32	6.65	3	0	80
GIN	1539	4.71	6.82	2	0	80
EIM	1539	3.99	9.55	0	0	153
ME.385						
Age (days)	1214	385.66	23.60	388	339	428
BW (kg)	1214	238.87	35.44	237	135	411
TICK	1214	9.09	11.34	5	0	131
GIN	1214	4.93	6.33	3	0	43
EIM	1214	4.50	14.18	0	0	255
ME.443						
Age (days)	1546	443.18	23.69	446	390	485
BW (kg)	1546	261.21	36.29	260	156	380
TICK	1546	5.34	7.36	3	0	63
GIN	1546	5.79	7.64	3	0	80
EIM	1546	3.45	13.27	0	0	284
ME.498						
Age (days)	1458	498.23	23.76	501	446	541
BW (kg)	1458	305.67	36.84	306	176	429
TICK	1458	6.24	8.27	3	0	80
GIN	1458	5.13	6.28	3	0	71
EIM	1458	3.63	12.79	0	0	182
ME.555						
Age (days)	1550	555.22	23.52	558	501	597
BW (kg)	1550	337.27	37.91	336	214	467
TICK	1550	6.48	8.71	3	0	72
GIN	1550	4.22	6.20	2	0	73
EIM	1550	3.30	13.91	0	0	328

*n* number of observations, *sd *standard deviation, *min* minimum value, *max* maximum value

The bulls included in the present study were subjected to natural parasite infestation. Prophylactic parasite control is a routine strategy on the farm and integrates a group of sanitary management practices. In the studied herd, this strategy includes deworming with Ivermectin 4% (1 mL of Ivermectin per 50 kg of live BW—Master LP, Ouro Fino Saúde Animal, Cravinhos, SP) at the beginning of the performance tests (day 1 of the adaptation period). Approximately 65% of the bulls were dewormed. The choice of animals that received treatment was based on contemporary groups in such a way that all the animals that belonged to randomly chosen contemporary groups were dewormed.

Blood samples were collected with sterilized syringes into 3.5-mL vacuum tubes containing 9NC coagulation sodium citrate 3.2%, to prevent blood from clotting and maintain DNA integrity. Blood samples were frozen and transferred into chilled coolers in the laboratory and stored in freezers at − 20 °C. In total, 1230 blood samples were selected for genotyping with a low-density DNA array, i.e. the Z-chip v2 (Neogen, Lincoln, Nebraska, EUA, which contains 27,533 single nucleotide polymorphisms (SNPs) mapped to the ARS-UCD1.2 bovine genome assembly). Most of the genotyped bulls were from the performance tests with more than 20 animals per group, as described above, and each animal had data for the three parasites for at least four ME.

The quality control of DNA samples and markers was carried out using the SNP & Variation Suite v8.8.3 software [16]. Alleles with a GenTrain Score < 0.6 were considered as missing calls in the panel. Only SNPs with a call rate ≥ 0.95, a minor allele frequency ≥ 0.05, and located on the autosomes and the X chromosome, and samples with a call rate > 0.90 were kept for further analyses. After quality control, the SNP panel included 21,667 SNPs (78.7% of all tested SNPs) and 1075 samples (87.4% of genotyped samples).

### Covariance components

We used single-trait linear random regression models (STM) where BW at each ME was considered as a dependent variable (trait) and the median count of the parasites for each cohort (Table 2) as an independent variable and no effect of co-infection, therefore we generated 15 datasets with both BW records and parasite counts. In the remainder of this paper, environmental parasite burden will refer to the median count of parasites for each cohort, which is our proxy for the strength of external (environment) infection, and not for individual parasite burden.

**Table 2:** Summary description of the median counts of ticks (TICK), gastrointestinal nematodes (GIN), and *Eimeria* spp. (EIM) per cohort

Trait per cohort	n	Median	Min	Max
ME.331				
TICK	48	3.50	0	16
GIN	48	3.25	0	9
EIM	48	0.00	0	11
ME.385				
TICK	40	7.00	0	33
GIN	40	4.00	0	11
EIM	40	2.00	0	10.50
ME.443				
TICK	48	2.75	0	15
GIN	48	4.00	0	12
EIM	48	0.00	0	16.50
ME.498				
TICK	44	4.00	0	16
GIN	44	3.00	1	8
EIM	44	0.00	0	11
ME.555				
TICK	48	4.25	0	18
GIN	48	2.75	0	8
EIM	48	0.00	0	7

*n* number of cohorts, *Min and Max* minimum and maximum median counts used to describe each cohort, respectively

The 15 STM were implemented using the Bayesian inference methodology and can be described as:

$${\text{y}}_{\text{ijkl}}={\text{c}}_{\text{j}}+{\text{d}}_{1}{\text{m}}_{(\text{k})}+{\text{b}}_{0}+{\text{b}}_{1}{\text{x}}_{\left(\text{l}\right)}+{\text{a}}_{{0}_{(\text{i})}}+{\text{a}}_{{1}_{\left(\text{i}\right)}}{\text{x}}_{(\text{l})}+{\text{e}}_{\text{ijkl}},$$

where ${\text{y}}_{\text{ijkl}}$ is the weight of the animal $\text{i}$, evaluated for cohort $\text{j}$, at age $\text{k}$ and submitted to an environmental parasite burden $\text{l}$; ${\text{c}}_{\text{j}}$ is the systematic effect of cohort $\text{j}$; ${\text{d}}_{1}$, is the slope to fit the effect of age at which each animal was evaluated; ${\text{m}}_{(\text{k})}$ is the age (in days) of the animals on the day of evaluation; ${\text{b}}_{0}$ and ${\text{b}}_{1}$ are the intercept and slope to fit the BW mean trajectory along the parasite burden, respectively; ${\text{x}}_{(\text{l})}$ is the median of parasite counts (TICK or GIN or EIM) of the animals’ cohort; ${\text{a}}_{{0}_{(\text{i})}}$ and ${\text{a}}_{{1}_{(\text{i})}}$ are the random intercept and slope to fit the additive genetic effect of each animal $\text{i}$, respectively; and ${\text{e}}_{\text{ijkl}}$, represents the error associated with each observation. It is important to highlight that ${\text{a}}_{{0}_{(\text{i})}}$ estimates the genetic effects for BW in the absence of parasite challenge, and ${\text{a}}_{{1}_{(\text{i})}}$is the HR. Furthermore, we assumed that there is a covariance between ${\text{a}}_{{0}_{(\text{i})}}$ and ${\text{a}}_{{1}_{(\text{i})}}$ and the variance components associated with those effects were estimated using pedigree information (traditional BLUP).

The additive genetic variances for BW for each observed environmental burden were estimated by the product $\mathbf{P}\otimes \mathbf{G0}\otimes \mathbf{P}^\mathbf{{\prime}}$. The $\mathbf{P}$ matrix has a number of lines equal to the number of different environments and two columns. The first column of $\mathbf{P}$ is a vector of 1s for adjusting the intercept, and the second column is the vector containing the observed median of parasite burden in the different cohorts; $\mathbf{P}^\mathbf{{\prime}}$ is the transpose of the $\mathbf{P}$ matrix; $\mathbf{G0}$ is the covariance matrix between the regression coefficients ${\text{a}}_{{0}_{(\text{i})}}$ and ${\text{a}}_{{1}_{(\text{i})}}$, and $\otimes$ is the Kronecker product. For further information about the STM see Additional file 3: Methods.

### Genome-wide association studies

The 20 (4 traits × 5 ME) genome-wide association studies (GWAS) were carried out for HR to the three parasites and for BW and five ME using the SNP & Variation Suite v8.8.3 software [16]. A mixed model was used to estimate the solutions for each of the 21,667 SNPs that passed quality control and an association test *P-value* related to each SNP solution was generated. For the GWAS, we used genotypes from animals for which their samples passed quality control (1075 samples). For all these animals, both BW records and estimated breeding values (EBV) for HR were available. The GWAS model used for BW can be described as:

$$\mathbf{BW}=\mathbf{Xb}+\mathbf{Zu}+\mathbf{Sa}+\mathbf{e},$$

where $\mathbf{BW}$ is the vector of body weight records for each animal at each evaluated age; $\mathbf{X}$ is the incidence matrix for the fixed covariates (cohort and age); $\mathbf{b}$ is the vector of solutions for the fixed effects; $\mathbf{Z}$ is an incidence matrix for the genetic additive random effects (estimated from the GRM); $\mathbf{u}$ is the vector of the solutions for the random additive genetic effects related to the observations; $\mathbf{S}$ is a matrix of genotypes (coded as 0, 1, or 2 copies of minor allele) for the evaluated SNPs, with number of rows equal to the number of genotyped animals, and number of columns equal to the number of SNPs in the genotype panel; $\mathbf{a}$ is the estimated effect of the evaluated SNP; and $\mathbf{e}$ is the vector of errors associated with each observation. SNP & Variation Suite v8.8.3 [16] uses a restricted maximum likelihood to estimate the solutions for the unknown parameters of the model.

Estimated breeding values estimated for HR to each parasite at each ME using STM were considered as the pseudo-phenotypes of HR for GWAS with no additional fixed effect. Note that using EBV as phenotypes may lead to double-counting of information and heterogeneous residuals. Given that all animals in the analysis had own phenotypes, it is expected that they had reasonably similar EBV, and thus homogeneous residuals, and that the double-counting of information was limited. Thus, the model used for GWAS for HR can be described as:

$$\mathbf{HR}=\mathbf{Zu}+\mathbf{Sa}+\mathbf{e},$$

where $\mathbf{HR}$ is the vector of EBV for host resilience to TICK, GIN, or EIM at each ME and the other terms are as previously described. The GRM used for GWAS was calculated according to the first method of VanRaden [17]. We applied it with a full dosage compensation correction to include the markers on the X chromosome in the calculation of the GRM since the number of copies of the alternate allele at any locus of X-chromosome can only be zero or one, which makes allele frequency calculations for SNPs at X-chromosomes in males different from the calculation of allele frequencies at autosomes [18]. It is not our objective to discuss about the effect of dosage compensation on the evaluated traits. However, there is evidence of this effect on the variation of complex phenotypes [19], thus we proceeded with the dosage compensation correction. Further information about the topic is described in Sidorenko et al. [19].

It is important to highlight that, in the present study, the heritability for BW was estimated based on both the GRM and the pedigree-based relationship matrix, thus for BW we present both the conventional heritability and the SNP-derived heritability estimates.

The pairwise SNP correlations of BW with HR to TICK, HR to GIN, and HR to EIM for each ME were computed by the Pearson correlation between the SNP effects of each one of the traits, as proposed by Fortes et al. [20], and will be considered in the present paper as proxies for the genetic correlations between traits.

### Quantitative trait loci associated with host resilience

The sample-size-based approach described by Willer et al. [21] was used to perform the meta-analysis to combine the results of the five described GWAS of HT to TICK (GIN and EIM) using the SNP & Variation Suite v8.8.3 [16]. In summary, a Z-score and an overall *P-value* for each marker were calculated by combining the SNP *P-value*, direction of the effect, and sample size generated by the previous GWAS. The meta-analysis was performed for markers that had solutions estimated in at least two studies (i.e. for at least two ME) and no genomic control was performed during the meta-analyses. Then, we used a Bonferroni correction to define the two groups of SNPs: those that were significantly associated with a *P-value* < 2.31* × *10^−6^ and those that were suggestively associated with a *P-value* < 10^−4^ [22, 23].

Based on the results of the meta-analysis, we defined quantitative trait loci (QTL) associated with each trait. The QTL boundaries were defined as follows: first, we identified an initial peak, i.e. the SNP with the lowest *P-value* for each chromosome (Chr); second, we searched for significant SNPs within 0.5-Mbp regions up and downstream of the peak SNP. If we identified other significative SNPs within this interval, the boundaries of the QTL were expanded to include the SNP and another 0.5-Mbp region (up and downstream) was investigated. The process was repeated until there was no more significant SNPs in these 0.5-Mbp windows. Finally, a new peak SNP was called if there was a significant SNP on the same Chr but outside of the boundaries of the first QTL. The process was repeated for each Chr until no more peak SNPs could be identified.

Moreover, only regions with at least four significant or suggestive SNPs were considered as QTL (adapted from van den Berg et al. [24]). In addition, only suggestive SNPs (*P-value* < 10^−4^) that were in high linkage disequilibrium (LD) with the peak or another significant SNP in the QTL were considered. The LD between SNPs was evaluated by the D prime (D’) value that was estimated using the expectation–maximization method by pairwise analysis in the SNP & Variation Suite v8.8.3 [16]. SNPs were considered in high LD when D′ was greater than the mean + 2 standard deviations of the D′ computed between all combinations of SNPs on the same Chr.

We searched for genes located within the QTL boundaries using the ARS-UCD1.2 bovine genome assembly (available at https://www.ncbi.nlm.nih.gov/assembly/GCA_002263795.2) with the GALLO package [25] of the R software [26]. This process resulted in a list of target candidate genes for HR to each parasite, which were used for the candidate gene prioritization analysis.

Candidate gene prioritization analysis was conducted using the ToppGene Suite [27] and consisted of two-steps. First, for each trait, a functional enrichment analysis was performed by building a list of the genes that were more likely to be related with our phenotypes, hereafter named the trained gene list. This trained gene list was constructed based on keywords (see Additional file 1: Table S2) that describe each of the evaluated phenotypes (BW and HR to the three parasites). These lists were obtained using the web application GUILDify v2.0 [28] for the phenotypic characterization of genes. GUILDify searches for genes starting from user-provided keywords in the Biologic Interaction and Network Analysis (BIANA) knowledge database. The genes associated with the keywords are used as seeds to generate the protein interaction networks, for the selected organism, and analysed with graph theory algorithms to prioritize new disease genes [28]. In the present study, the selected model organism was *Homo sapiens*, since bovine was not an option. The Netscore prioritization algorithm from the GUILD package was used (with repetition = 3 and interaction = 2; default values of GUILDify). The output of GUILDify is a trained list of genes that are ranked according to the interaction network. The first 100 genes were used as the trained gene list for each studied trait.

For functional enrichment analysis, the trained gene list was compared with random sets of genes in the genome to search for any functional category or parameter that was overrepresented in our trained list compared with the background. We used Gene Ontology (Molecular function, Biological process, and Cellular component), Human phenotype, Mouse phenotype, Pathway, PubMed, Transcription factor binding site, Co-expression, and Disease as training databases. The *P-value* cut-off for each training parameter was 0.05 with a false discovery rate correction. After this step, a representative profile of the trained gene list was obtained.

In the second step, a similarity score was generated for each gene in our list of candidate genes. This score is created by functional annotation of the candidate gene followed by a comparison of its function to each enriched term that is learned in the training step. The similarity score calculation and the associated *P-values* are described in Chen et al. [27]. In summary, a fuzzy-based similarity measure is applied for categorical terms [29], and Pearson correlation between the test gene and the enriched gene lists is applied for quantitative functional parameters. In the case of a missing value (for instance, lack of one or more annotations for a test gene), the score is set to − 1. Otherwise, it is a real value within [0, 1] [27]. At the end of this process, each gene will have one similarity score and one *P-value* for each one of the functional categories.

A final test is carried out to compute an overall *P-value* that will be used to determine if each gene was prioritized or not. For this computation, we need to assume that the *P-values* come from independent tests, thus the Fisher’s inverse chi-square method was applied to combine the *P-values* from multiple annotations into an overall *P-value* [27]. The prioritized genes were considered to be those with an overall *P-value* ≤ 0.05. For the candidate gene prioritization analysis, we used the default setting in the ToppGene Suite that has a background gene set from the genome for computing the *P-value* with 5000 coding genes and two features to be considered for prioritization.

## Results

### Genetic parameters for body weight and host resilience

In general, the highest posterior density interval with 90% of samples (HPD90) related to the posterior mean of the intercept and slope variances were wide, indicating no difference between genetic parameters estimated across ME. However, residual variances of BW estimated at ME.331 were smaller than those at ME.555 (Table 3). The posterior means of the correlation between intercept and slope were negative, but the HPD90 associated to those estimates were wide and included zero (Table 3).

**Table 3:** Posterior means of genetic parameters (limits of HPD90) for intercept (int) and slope coefficients of body weight at five measurement events (ME) when ticks (TICK), gastrointestinal nematodes (GIN), and *Eimeria* spp. (EIM) burden^a^ were used as independent variables in single-trait linear random regression models

ME	${\sigma}_{int}^{2}$	${\sigma}_{slope}^{2}$	${r}_{{int} \times {slope}}$	${\sigma}_{e}^{2}$
TICK				
331	186.15 (108.00; 262.40)	1.31 (0.29; 2.19)	− 0.90 (− 1.00; − 0.78)	360.51 (320.70; 401.60)
385	81.05 (9.92; 144.50)	0.32 (0.03; 0.59)	− 0.29 (− 0.92; 0.52)	465.09 (413.70; 518.30)
443	112.68 (27.18; 194.80)	0.95 (0.02; 1.84)	− 0.54 (− 1.00; − 0.01)	467.66 (416.00; 517.80)
498	145.38 (54.55; 231.10)	1.09 (0.11; 2.03)	− 0.45 (− 0.97; 0.03)	527.59 (465.00; 588.40)
555	126.41 (43.78; 214.20)	1.03 (0.06; 1.95)	0.02 (− 0.65; 0.87)	535.91 (470.80; 599.80)
GIN				
331	163.83 (70.57; 256.00)	4.53 (1.59; 7.67)	− 0.66 (− 0.91; − 0.45)	341.20 (297.80; 390.60)
385	188.58 (64.33; 301.40)	4.05 (0.97; 6.87)	− 0.74 (− 0.94; − 0.54)	439.23 (380.90; 496.90)
443	119.87 (17.18; 213.50)	1.68 (0.06; 3.10)	− 0.53 (− 1.00; 0.08)	468.77 (419.80; 519.40)
498	222.44 (8.12; 405.60)	6.86 (0.39; 12.92)	− 0.60 (− 0.98; − 0.17)	529.12 (468.70; 590.20)
555	69.36 (1.52; 130.10)	4.68 (0.56; 8.57)	0.18 (− 0.50; 1.00)	549.7 (485.80; 610.40)
EIM				
331	105.77 (38.78; 165.40)	2.65 (0.49; 4.51)	− 0.33 (− 0.83; 0.14)	341.27 (291.70; 385.20)
385	161.31 (57.58; 256.30)	8.16 (2.82; 13.50)	− 0.75 (− 0.96; − 0.53)	431.66 (375.00; 489.60)
443	74.85 (18.39; 131.30)	2.23 (0.42; 3.92)	− 0.07 (− 0.80; 0.72)	462.04 (412.00; 513.10)
498	101.27 (23.98; 172.60)	3.51 (0.62; 6.42)	− 0.07 (− 0.70; 0.57)	530.03 (470.80; 591.10)
555	192.40 (94.15; 292.50)	7.51 (0.96; 12.97)	− 0.52 (− 0.92; − 0.14)	536.81 (469.80; 605.00)

${\sigma }_{int}^{2}$ = additive genetic variance for the intercept; ${\sigma }_{slope}^{2}$ = additive genetic variance for the slope; ${r}_{int x slope}$ = genetic correlation between intercept and slope; ${\sigma }_{e}^{2}$ = residual variance

ME: measurement events when the age of animals was 331, 385, 443, 498 and 555 days on average

^a^Parasitic burden was modelled using information about the median infestation per cohort (contemporary group)

**Fig. 2:**
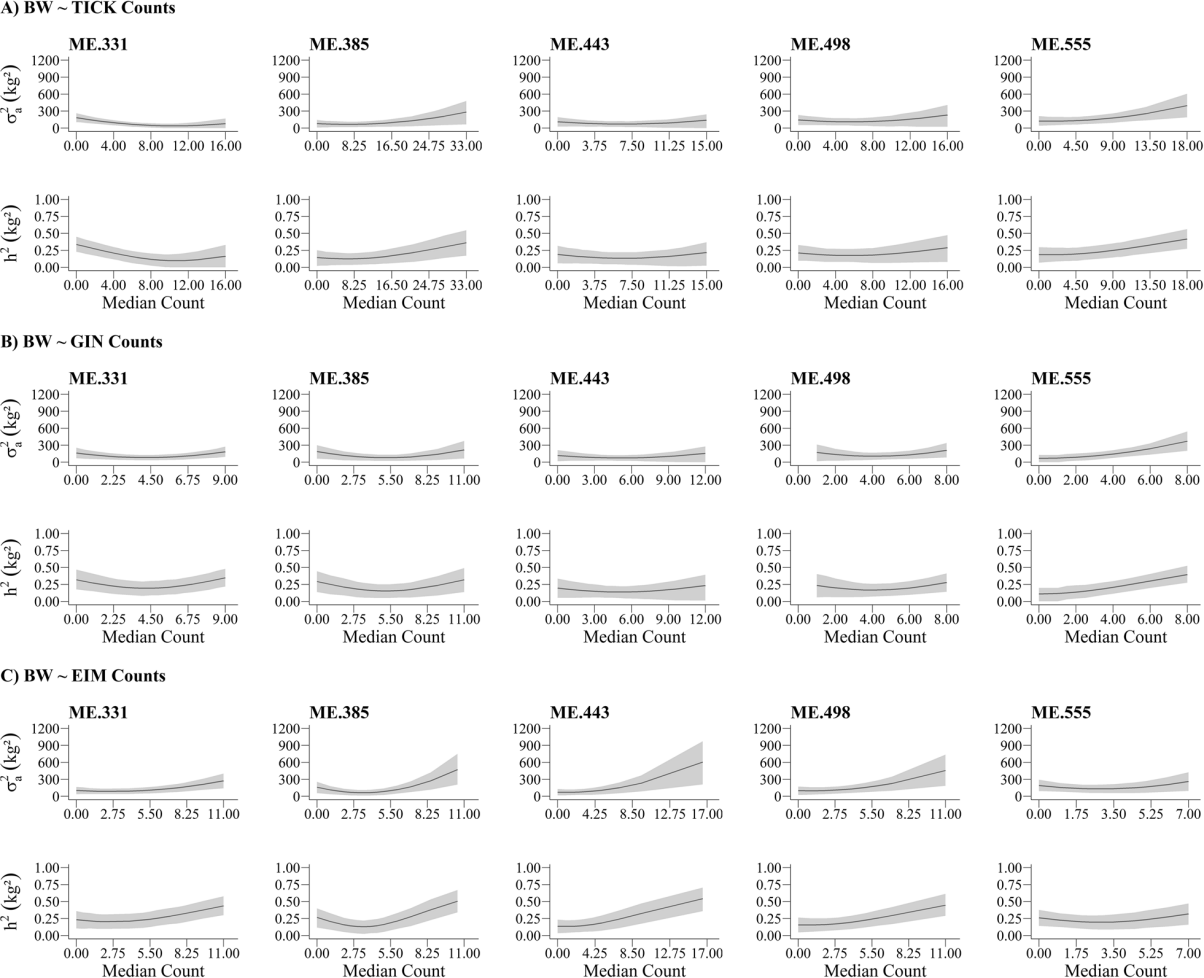
Additive genetic variances [${\upsigma}_{\text{a}}^{2}$ (kg^2^)] and heritability estimates (*h*^2^) for body weight (BW) across the trajectories of tick (TICK), nematodes (GIN), or *Eimeria* ssp. (EIM) burden at five measurement events (ME). ME.331, ME.385, ME.443, ME.498, ME.555 are body weights at each measurement event when the average age of animals was 331, 385, 443, 498 and 555 days, respectively

A rising trend for the additive variance and heritability of BW was observed across the trajectories of TICK, GIN, and EIM burden (Fig. 2). For instance, the posterior means for the heritability of BW ranged from 0.09 to 0.44 at ME.331, from 0.13 to 0.51 at ME.385, from 0.13 to 0.54 at ME.443, from 0.16 to 0.45 at ME.498 and from 0.11 to 0.42 at ME.555. In spite of the differences between the heritability estimates for BW when parasite count was zero and maximum (maximum count of 16 for TICK, 11 for GIN and 10.5 for EIM), the HPD90 related to these posterior means were large showing no significant differences between them (Fig. 2).

The SNP-derived heritability (average ± standard error) for BW (Table 4) at each ME ranged from a low (0.09 ± 0.06 at ME.331) to a moderate magnitude (0.23 ± 0.06 at ME.555), showing that genetic improvement of BW can be achieved through selection. Moreover, these values were similar to the heritability of BW estimated by STM (Fig. 2).

**Table 4:** SNP-derived heritability estimates (standard error) for body weight (BW) and host resilience to ticks (HR.TICK), gastrointestinal nematodes (HR.GIN) and *Eimeria* spp. (HR.EIM) at different measurement events (ME)

Trait	ME.331	ME.385	ME.443	ME.498	ME.555
BW	0.16 (0.06)	0.09 (0.05)	0.16 (0.05)	0.19 (0.06)	0.23 (0.06)
HR.TICK	0.81 (0.04)	0.87 (0.04)	0.81 (0.04)	0.87 (0.03)	0.76 (0.04)
HR.GIN	0.84 (0.04)	0.93 (0.03)	0.80 (0.04)	0.84 (0.04)	0.85 (0.04)
HR.EIM	0.79 (0.04)	0.82 (0.04)	0.77 (0.04)	0.80 (0.04)	0.84 (0.03)

ME.331, ME.385, ME.443, ME.498, ME.555: measurement events when the age of animals was 331, 385, 443, 498 and 555 days on average

The SNP-derived heritability estimates for HR to TICK, GIN, and EIM at each ME were computed through GWAS when the slope solutions (genetic effects) were considered as the HR phenotype. As expected, the magnitude of these estimates was large (Table 4), ranging from 0.76 to 0.87 for HR to TICK, from 0.80 to 0.93 for HR to GIN, and from 0.77 to 0.84 for HR to EIM.

**Fig. 3:**
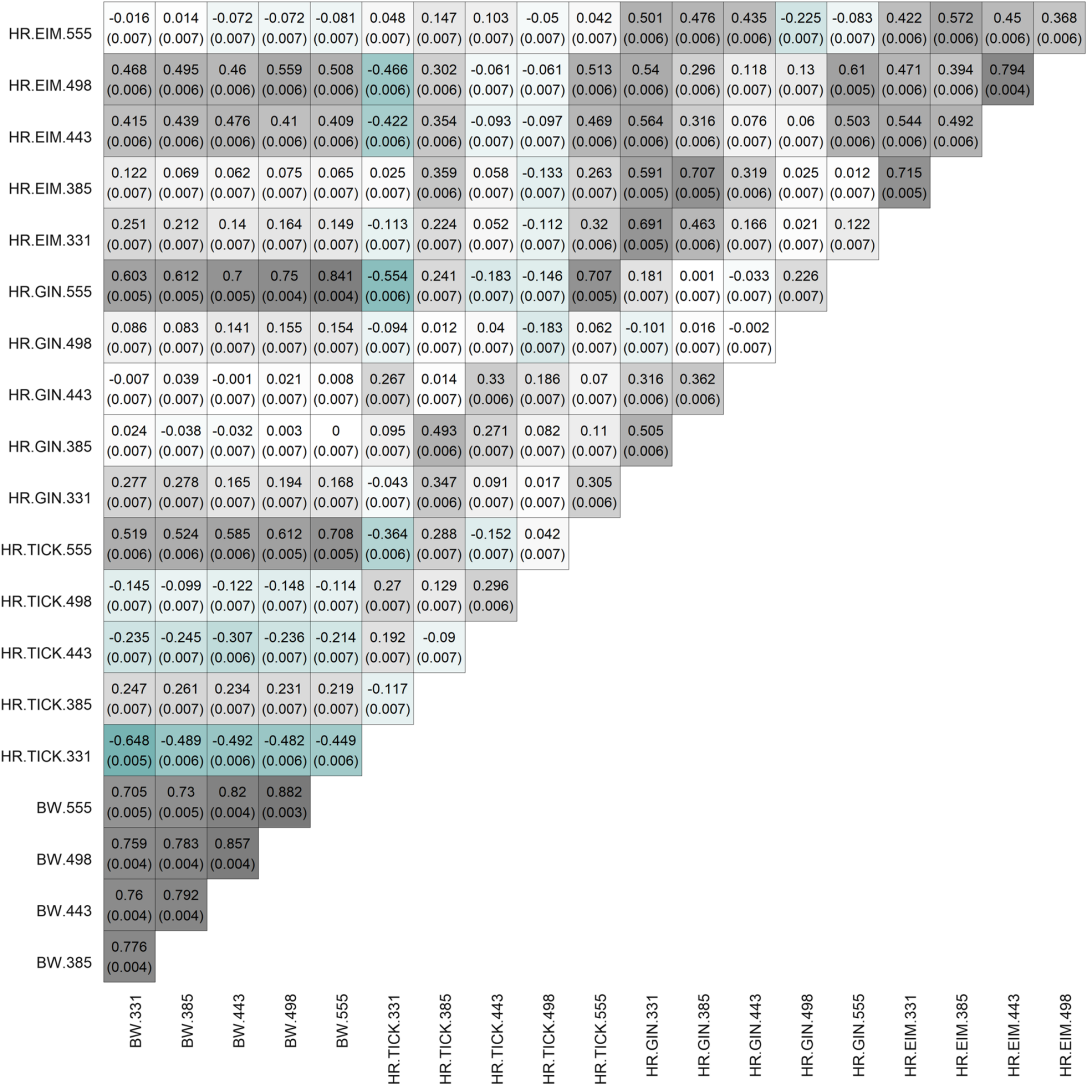
Pairwise SNP correlations between body weight (BW), host resilience to ticks (HR.TICK), gastrointestinal nematodes (HR.GIN), and *Eimeria* spp. (HR.EIM) measured at five measurement events (ME) when the average age of animals was 331, 385, 443, 498, and 555 days. The values above the diagonal are the Pearson correlations between SNP effects (and standard errors of SNP correlations)

While in some cases, the genetic correlations between intercept and HR did not differ from zero (because HPD90 includes a zero value), the pairwise SNP correlations indicate that there is some genetic association between BW and HR. In general, the pairwise SNP correlations of BW with HR to GIN and HR to EIM were zero, or favourable (Fig. 3). However, pairwise SNP correlations of BW with HR to TICK at ME.331 (− 0.648 ± 0.005), ME.443 (− 0.307 ± 0.006), and ME.498 (− 0.148 ± 0.007) were unfavourable (Fig. 3). These correlations agree with those estimated between intercept and slope, that were also negative (Table 3).

### Candidate genes and pathways associated with host resilience to TICK, GIN, and EIM

**Fig. 4:**
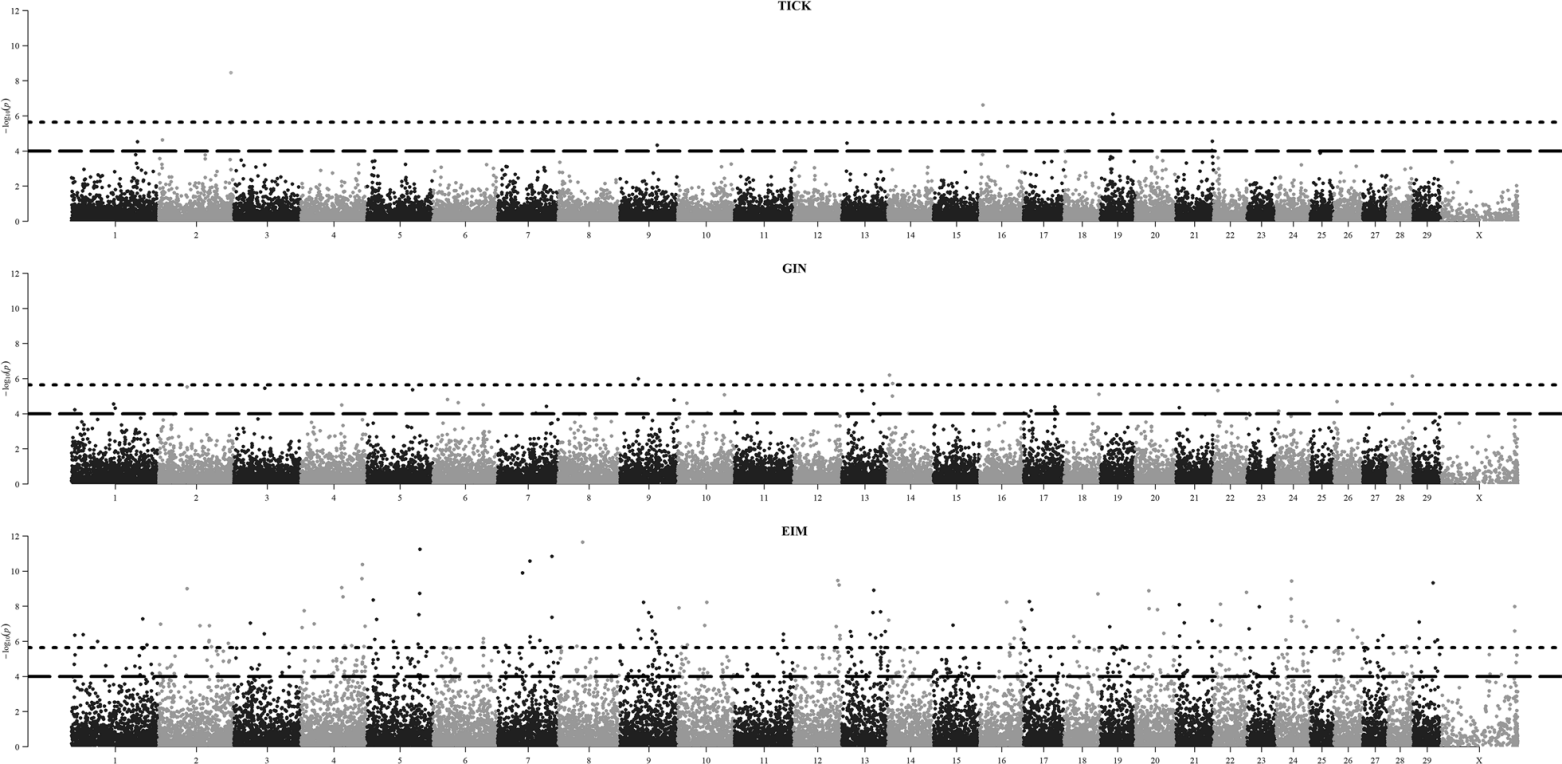
Manhattan plots for the meta-analysis of the genome-wide association studies for HR to ticks (TICK), gastrointestinal nematodes (GIN), and *Eimeria* spp. (EIM) measured at different measurement events. The dotted line (y = 5.64) indicates the threshold for statistical significance. The dashed line (y = 4.00) indicates the threshold for suggestive evidence of association

The genes that were associated with HR were searched within the QTL that were built from the significant and suggestive SNPs obtained by the meta-analysis GWAS (Fig. 4). The meta-analysis was processed using the GWAS results related to each age, separately, which are presented in Additional file 4: Fig. S2, Additional file 5: Fig. S3, and Additional file 6: Fig. S4. Information on the number of SNPs and linkage disequilibrium thresholds used to define the QTL boundaries are in Table 5.

**Table 5:** Description of the QTL associated with host resilience to *Eimeria* spp.

Chr	N_SNP_	N_sigSNP_	N_sugSNP_	LD_CHR_	sd_LD_	LD_1−n_
4	11	1	3	0.17	0.19	0.70
6	14	2	2	0.15	0.16	0.51
7	11	3	1	0.15	0.16	0.85
12	17	3	1	0.17	0.17	0.71
13	39	2	6	0.15	0.16	0.54

Chr: chromosome; N_SNP_: number of SNPs inside the QTL; N_sigSNP_: number of significant SNPs inside QTL (associated *P-values* < 2.31* × *10^−6^); N_sugSNP_: number of suggestive SNPs inside QTL (associated *P-values >* 2.31* × *10^−6^ and *P-values* < 2.31* × *10^−4^); LD_CHR_: average linkage disequilibrium observed between SNPs of each Chr; sd_LD_ = standard deviation of LD_CHR_; LD_1−n_: linkage disequilibrium between the first and last SNP of a QTL

Apart from the presence of some significant isolated SNPs, we detected no QTL, i.e. no genomic regions, that were associated with HR to TICK and HR to GIN. Five QTL located on Chr 4, 6, 7, 12, 13 were associated with HR to EIM (Table 6). In total, 47 genes were located within these QTL regions (Table 6) and among these, 16 were prioritized. Information about the genes that were prioritized for HR to EIM is in Additional file 1: Table S3.

**Table 6:** Description of the quantitative trait locus (QTL) defined from SNPs that were significantly associated with host resilience to *Eimeria* spp.

Chr	n	IP	FP	Genes inside QTL
4	11	116,439,784	117,037,674	*DPP6*^a^, *HTR5A*^a^, *PAXIP*^b^, *RF00006*^c^
6	14	90,646,323	91,785,192	*CXCL9*^a^, *CXCL10*^a^, *CXCL11*^a^, *NAAA*^a^, *SCARB2*^a^, *STBD1*^a^, *ART3*^b^, *CCDC158*^b^, *NUP54*^b^, PPEF2^b^, *SDAD1*^b^, *SHROOM3*^b^, *SOWAHB*^b^, *ENSBTAG00000004921*^c^, *ENSBTAG00000032074*^c^, *ENSBTAG00000050665*^c^, *ENSBTAG00000053885*^c^, *ENSBTAG00000054432*^c^, *SEPT11*^c^, *RF00003*^c^, *RF00026*^c^
7	11	58,461,990	59,477,630	*DPYSL3*^a^, *SPINK1*^a^, *SPINK5*^a^, *JAKMIP2*^b^, *SCGB3A2*^b^, *SPINK6*^b^, *STK32A*^b^, *bta-mir-2284y-7*^*c*^, *C7H5orf46*^*c*^, *ENSBTAG00000052309*^c^, *ENSBTAG00000053960*^c^, *RF00026*^c^
12	17	83,457,070	84,943,864	*COL4A1*^a^, *IRS2*^a^, *LIG4*^a^, *TNFSF13B*^a^, *ABHD13*^b^, *MYO16*^b^, *RF00001*^c^
13	39	70,341,842	71,369,326	*PTPRT*^a^, *ENSBTAG00000002446*^c^, *RF00026*^c^

Genes marked with ^a^were prioritized in the candidate gene prioritization analyses, ^b^were included in the analyses but not prioritized, and genes with ^c^were not included in the prioritization analyses

*Chr* chromosome, *n* number of SNPs within the QTL, *IP* initial position, *FP* final position

## Discussion

### Environmental parasite burden

In our study, the median counts of parasites were used as environmental gradient, once we considered it the best descriptor of parasitic load challenging a group of contemporary animals. First, it is important to mention that the environmental and animal loads are highly dependent due to the fact that life cycle of parasites involves a period of time spent in a host organism. For instance, the adult tick females lay their eggs in the pasture, where hatching occurs. Larvae, nymphs, and adults parasitize the host through feeding, and then drop off again on the pasture to continue their life cycle [4]. Similarly, gastrointestinal parasites and *Eimeria* spp. have multiple stages of development that include some time spent in the host organism but not their entire life cycle [30, 31]. However, individual loads depend on factors, such as animal resistance, behaviour, and other individual factors, and, thus, using individual parasitic loads can mask the real environmental challenges. For instance, more resistant animals might be within cohorts that are highly challenged and even then, present zero parasite counts. The opposite is also true, when exposed to low environmental loads, a highly susceptible animal might face a high parasitic load, although most of its contemporaries are parasite free.

HR, which is the main object of our study, is better estimated when environmental parasitic loads are available [32]. Such measurements were not available since we worked with data from a commercial herd, which means that the animals were not submitted to a high and artificially infested environment. Thus, we used the parasite counts observed in different animals from the same cohort as an indicator of the environmental load. Since counts are discrete measurements, we feel that using medians instead of average counts would better describe the common load to which all animals from a same cohort were exposed. It is important to highlight that we developed this study based on environmental parasitic burden, which is a proxy for environmental infection pressure (or strength of environmental infection), and not for host parasitic burden.

At least one animal in each cohort had parasite counts greater than zero, even in cohorts for which median parasite counts are zero, therefore there were no parasite-free cohorts and every single animal in our dataset was exposed to natural infestation of TICK, GIN and EIM. A zero environmental load only indicates that the animals in these cohorts are less challenged than those in cohorts with a median parasite load equal to 5, for example. The observed median counts reported here are similar to those of other datasets in crossbreed Angus and Nellore [33], Colombian *Bos taurus* cattle breeds [34], and German Black and White dairy cows [35]. The low parasite load observed here might be partially explained by different factors, including the breed evaluated. Nellore cattle is an indicine breed known to be more resistant to highly infested environments [36, 37]. In addition, the adoption of rotational grazing [38], and prophylactic parasite control strategies largely applied in commercial herds might contribute to low parasite loads. It is important to have in mind that such controlled parasite burden through prophylactic treatment, as in this study, represents the reality on commercial farms [39–41].

### Genetic parameters for body weight and host resilience

The SNP-derived and STM heritability estimates for BW obtained here were similar to those reported previously for the same population [42]. These results confirmed that the low-density SNP panel (27K—Z-chip V2, Neogen, Lincoln, Nebraska, EUA) can capture the polygenetic component of the additive variance observed for BW in Nellore cattle. The heritability estimates for BW in this population were lower than those reported in the literature for Nellore cattle of similar ages raised in Brazil, using pedigree information only [43], which can be partially explained by the fact that the studied population is under selection. Selective breeding can lead to lower genetic variability, and consequently, lower heritability estimates [44]. The selective breeding program from Mundo Novo farm was implemented in 1978 without including external candidates, with an intense selection for BW, and an efficient animal husbandry approach that corresponds to the outstanding practices of a nucleus farm. Regarding the high SNP-derived heritabilities estimated for HR in the present study, they do not indicate that HR is highly heritable. In fact, these values are a statistical artefact since the phenotype of HR is itself an estimated breeding value. However, the SNP-derived heritability indicates that breeding values estimated using pedigree-based genetic evaluations can be efficiently explained by the genomic similarity between individuals.

In the present study, we observed a curved trajectory of the heritability estimates for BW as the parasitic loads increased, with an overall rising trend if we compare extreme environments only, however this increase was not significant since the HPD90 overlapped. Marques et al. [45] observed a rising trend of the heritabilities for faecal egg counts (FEC) and a reduction in the heritabilities for BW in Corriedale sheep between environments with low or high FEC, but as in our case, these differences were not significant because the HPD of heritability estimates overlapped. Challenging environments with higher natural or artificial parasite loads are expected to lead to more significant effects on both BW and the genetic parameters for HR [46]. For instance, the highest heritability estimates for the FAMACHA score in ram and ewe lambs were obtained in high worm burden scenarios [47]. Similarly, an uprise in the trend of heritability estimates for milk yield was observed with increased temperature-humidity index, a direct indicator of heat stress [48]. However, it is important to highlight that opposite trends for genetic variances and heritability estimates can be observed with increasing parasite burden. Hollema et al. [49] showed that a significant decrease in the heritability for growth rate of Australian Merino sheep with increasing worm burden. These authors argued that animals in an environment with a high worm burden were not able to show their genetic potential for growth in the same way than animals in an environment with a low worm burden could [49]. In short, parasite burden can affect animal performance with consequences for the heritability estimates.

The pairwise SNP correlations between BW at different ME and the genetic correlations between intercept and HR, indicate the presence of a genotype × parasite burden interaction for BW, which means that parasite burden might impact the EBV for BW, with consequences for selective breeding. It is important to consider parasite loads in selective breeding programs for BW and growth on pastured systems, especially in tropical areas. Varying levels of natural infestation, and different strategies for parasite control will impact animal performance and therefore affect genetic predictions as well. Thus, it is relevant to develop selection strategies that consider multiple breeding objectives, including phenotypes that might be indicators of animal health or parasite load, like the use of ImmuneDEX (IDEX) as selection criteria [50]. IDEX is an index that combines animal’s ability to mount a cell-mediated immune response (Cell-IR) and an antibody-mediated immune response and can help in the identification of immune competent animals.

The unfavourable correlations between BW and HR to TICK were stronger for younger (ME.331) than older animals (ME.550). This result might be partially explained by the effect of age on immune response mechanisms [51–53]. Moreover, the association between animal size, skin surface and vasculature density might influence these unfavourable correlations [34]. Complementary studies are necessary to investigate the genetic mechanisms that underlie HR to different parasites at different growth stages. Considering the varying impact of HR at different ages, selection programs that measure BW at different ages, can develop a selection index to target multiple traits with a balancing approach, which might do a better job than targeting only BW at a young age. On the opposite side, selection for HR to GIN and HR to EIM will either benefit to or have no impact on BW, in general. These findings need to be further studied and validated on larger populations, and with more extreme parasite loads.

### Candidate genes and pathways associated with host resilience

The methods, which were used here to search for functional candidate genes only in the QTL regions, filtered out the search for genes associated with HR to TICK and HR to GIN. In spite of the absence of significant QTL, some SNPs were significantly associated with these traits. For research purposes and with the main objective of avoiding the discussion of spurious associations from the GWAS, we believe that adding the QTL definition based on the presence of multiple significant and suggestive SNPs at a given region, is a good quality control to select for true associations. However, we do acknowledge that the population with available phenotypes was small, and that regions with significant SNPs associated with HR to TICK and HR to GIN might be within important QTL that were not identified because of the limitations due to the size of our database. Thus, future studies might bring other interesting insights on HR.

We identified several genes in the chemokine pathways, such as *CXCL9*, *CXCL10*, and *CXCL11*, which were related to HR to EIM. The transcripts of these genes are proinflammatory chemokines that are released from the intestinal epithelium [54]. The levels of both *CXCL9* and *CLCX11* transcripts were found to be increased in the gut tissue of susceptible mice that were artificially infected with *Trichuris muris*, but the up-regulation of these genes has not been verified in artificially-infected resistant mice [55]. Furthermore, in vivo neutralization of the *CXCL10* gene resulted in a significant reduction in worm burden and increased rate of epithelial cell turnover in infected susceptible mice [56]. Cliffe et al. [56] demonstrated that CXCL10 had no effect on the TH1 immune response of susceptible animals, indicating that epithelial cell turnover alone can mediate worm expulsion. The *CXCL9* gene plays an important role in antimicrobial defence by protecting the gut of artificially infected mice from the invasion by the bacteria *Citrobacter rodentium* and restoring the damaged tissue. These studies in mice suggest a possible mechanism underpinning the association of CXCL9, CXCL10, and CXCL11 with HR. The immune responses mediated by chemokines is probably an important mechanism for HR to all intestine parasites, including protozoans. However, further studies in cattle are necessary to confirm the role that these genes might play in HR.

The modulators of antibody-mediated immune response, i.e. the *IRS2*, *LIG4*, and *TNFSF13B* genes were associated to HR to EIM in our study. These genes were also associated with human susceptibility to the nematode *Ascaris lumbricoides*, an endemic disease in tropical areas [57]. The *TNFSF13B* gene plays an important role in the class-switch recombination process and in the proliferation of B cells by rearranging its DNA sequence to switch their expression from one class of immunoglobulin, such as IgM, to an immunoglobulin heavy-chain constant region, which results in antibodies with different effector functions [58]. Moreover, the expression of *TNFSF13B* in the intestinal tissue of chickens that were orally infected with *Eimeria acervulina* increased after coccidiosis infection and led to a high antibody response [59]. Therefore, expression of HR to EIM can be associated with intestinal homeostasis maintenance and adaptive immune response. The genes that are significantly associated with HR to EIM were previously associated with nematode infections thus, it is possible that the defence mechanisms developed by animals exposed to GIN and EIM are partially similar. Further studies are required to validate these associations and the possible mechanisms that link the above discussed candidate genes with HR in cattle.

## Conclusions

Selection under natural infestation and controlled parasite burden, via prophylactic parasite control, contributes to identify animals that are resilient to nematodes and *Eimeria* ssp. and that are expected to perform better under challenging environments (i.e. tropical regions). Chemokine pathways and intestinal epithelial cells are important for HR to gastrointestinal parasites and further studies focused on the expression of the candidate genes discovered in this study might help to better understand the HR mechanisms to different parasites.

### Supplementary Information

**Additional file 1: Table S1.** Number of repeated measurements per animal. **Table S2.** Keywords used to construct the trained list of genes for body weight (BW) and host tolerance to ticks (HT.TICK), gastrointestinal nematodes (HT.GIN) and *Eimeria* spp. (HT.EIM). **Table S3.** Summary statistics of genes submitted to candidate gene prioritization analysis for host tolerance to *Eimeria* spp.

**Additional file 2: Figure S1.** Distributions of body weight information (BW-**a**), ticks (TICK-**b**), gastrointestinal nematodes eggs (GIN-**c**), and *Eimeria* spp. oocysts (EIM-**d**) counts at each measurement event (ME). 331, 385, 443, 498, and 555 represent the mean ages of the animals at each ME, respectively.

**Additional file 3.** Additional information on the methods used to process the statistical analysis [60–65].

**Additional file 4: Figure S2.** Manhattan plots for the genome-wide association studies for host tolerance to ticks evaluated at different measurement events (ME). 331, 385, 443, 498, and 555 are the mean ages (in days) of the animals at each ME. The dotted line (y = 5.64) indicates the threshold for statistical significance. The dashed line (y = 4.00) indicates the threshold for suggestive evidence of association.

**Additional file 5: Figure S3.** Manhattan plots for the genome-wide association studies for host tolerance to gastrointestinal nematodes evaluated at different measurement events (ME). 331, 385, 443, 498, and 555 are the mean ages (in days) of the animals at each ME. The dotted line (y = 5.64) indicates the threshold for statistical significance. The dashed line (y = 4.00) indicates the threshold for suggestive evidence of association.

**Additional file 6: Figure S4.** Manhattan plots for the genome-wide association studies for host tolerance to *Eimeria* spp. evaluated at different measurement events (ME). 331, 385, 443, 498, and 555 are the mean ages (in days) of the animals at each ME. The dotted line (y = 5.64) indicates the threshold for statistical significance. The dashed line (y = 4.00) indicates the threshold for suggestive evidence of association.

## Data Availability

The datasets used and/or analysed during the current study are available from the corresponding author on reasonable request.
