# Supplementary material for: Unravelling the genetic variability of host resilience to endo- and ectoparasites in Nellore commercial herds
Source: Genet Sel Evol. 2023 Nov 21;55:81. doi: 10.1186/s12711-023-00844-9 (PMC10664541; doi:10.1186/s12711-023-00844-9)
Supplement: Supplementary file 1 — Additional file 1: Table S1. Number of repeated measurements per animal. Table S2. Keywords used to construct the trained list of genes for body weight (BW) and host tolerance to ticks (HT.TICK), gastrointestinal nematodes (HT.GIN) and Eimeria spp. (HT.EIM). Table S3. Summary statistics of genes submitted to candidate gene prioritization analysis for host tolerance to Eimeria spp. [file 12711_2023_844_MOESM1_ESM.docx]

Table S1. Number of repeated measurements per animal

| Number of measurements | Number of animals |
| --- | --- |
| 1 | 30 |
| 2 | 74 |
| 3 | 232 |
| 4 | 447 |
| 5 | 929 |

Table S2. Keywords used to construct the trained list of genes for body weight (BW) and host tolerance ticks (HT.TICK), gastrointestinal nematodes (HT.GIN) and *Eimeria* spp. (HT.EIM)

| Trait | GUILDify Keywords |
| --- | --- |
| BW | Body weight, Growth, Obesity, Protein, Muscle, Fat, Growth factors, Height |
| HT.TICK | Immunity, Immune response, Inflammation, Ectoparasite, Cytokines, Tick, Infection, Tolerance |
| HT.GIN | Immunity, Immune response, Inflammation, Endoparasite, Cytokines, Nematodes, Infection, Tolerance |
| HT.EIM | Immunity, Immune Response, Inflammation, Endoparasite, Cytokines, Eimeria, Infection, Tolerance |

Table S3. Summary statistics of genes submits to candidate genes prioritization analysis for host tolerance to *Eimeria spp*.

| Gene symbol | Gene name | Average score | Overall *P-value* |
| --- | --- | --- | --- |
| CXCL9 | C-X-C motif chemokine ligand 9 | 1.000 | 0.000 |
| CXCL10 | C-X-C motif chemokine ligand 10 | 1.000 | 0.000 |
| CXCL11 | C-X-C motif chemokine ligand 11 | 1.000 | 0.000 |
| TNFSF13B | TNF superfamily member 13b [ | 0.771 | 0.000 |
| SCARB2 | scavenger receptor class B member 2 | 0.718 | 0.001 |
| IRS2 | insulin receptor substrate 2 | 0.639 | 0.001 |
| SPINK5 | serine peptidase inhibitor Kazal type 5 | 0.727 | 0.001 |
| SPINK1 | serine peptidase inhibitor Kazal type 1 | 0.710 | 0.002 |
| HTR5A | 5-hydroxytryptamine receptor 5A | 0.607 | 0.005 |
| COL4A1 | collagen type IV alpha 1 chain | 0.547 | 0.005 |
| LIG4 | DNA ligase 4 | 0.598 | 0.006 |
| DPP6 | dipeptidyl peptidase like 6 | 0.618 | 0.007 |
| PTPRT | protein tyrosine phosphatase receptor type T | 0.563 | 0.015 |
| DPYSL3 | dihydropyrimidinase like 3 | 0.504 | 0.020 |
| NAAA | N-acylethanolamine acid amidase | 0.544 | 0.034 |
| STBD1 | starch binding domain 1 | 0.360 | 0.042 |
| NUP54 | nucleoporin 54 | 0.391 | 0.064 |
| SPINK6 | serine peptidase inhibitor Kazal type 6 | 0.389 | 0.084 |
| PAXIP1 | PAX interacting protein 1 | 0.378 | 0.099 |
| ART3 | ADP-ribosyltransferase 3 | 0.277 | 0.144 |
| PPEF2 | protein phosphatase with EF-hand domain 2 | 0.383 | 0.145 |
| SCGB3A2 | secretoglobin family 3A member 2 | 0.348 | 0.191 |
| MYO16 | myosin XVI | 0.274 | 0.222 |
| STK32A | serine/threonine kinase 32A | 0.270 | 0.224 |
| SHROOM3 | shroom family member 3 | 0.285 | 0.313 |
| JAKMIP2 | janus kinase and microtubule interacting protein 2 | 0.217 | 0.324 |
| SDAD1 | SDA1 domain containing 1 | 0.238 | 0.364 |
| SOWAHB | sosondowah ankyrin repeat domain family member B | 0.171 | 0.412 |
| ABHD13 | abhydrolase domain containing 13 | 0.110 | 0.500 |
| CCDC158 | coiled-coil domain containing 158 | 0.000 | 0.763 |
