# Supplementary figures and images for: Unravelling the genetic variability of host resilience to endo- and ectoparasites in Nellore commercial herds
Source: Genet Sel Evol. 2023 Nov 21;55:81. doi: 10.1186/s12711-023-00844-9 (PMC10664541; doi:10.1186/s12711-023-00844-9)

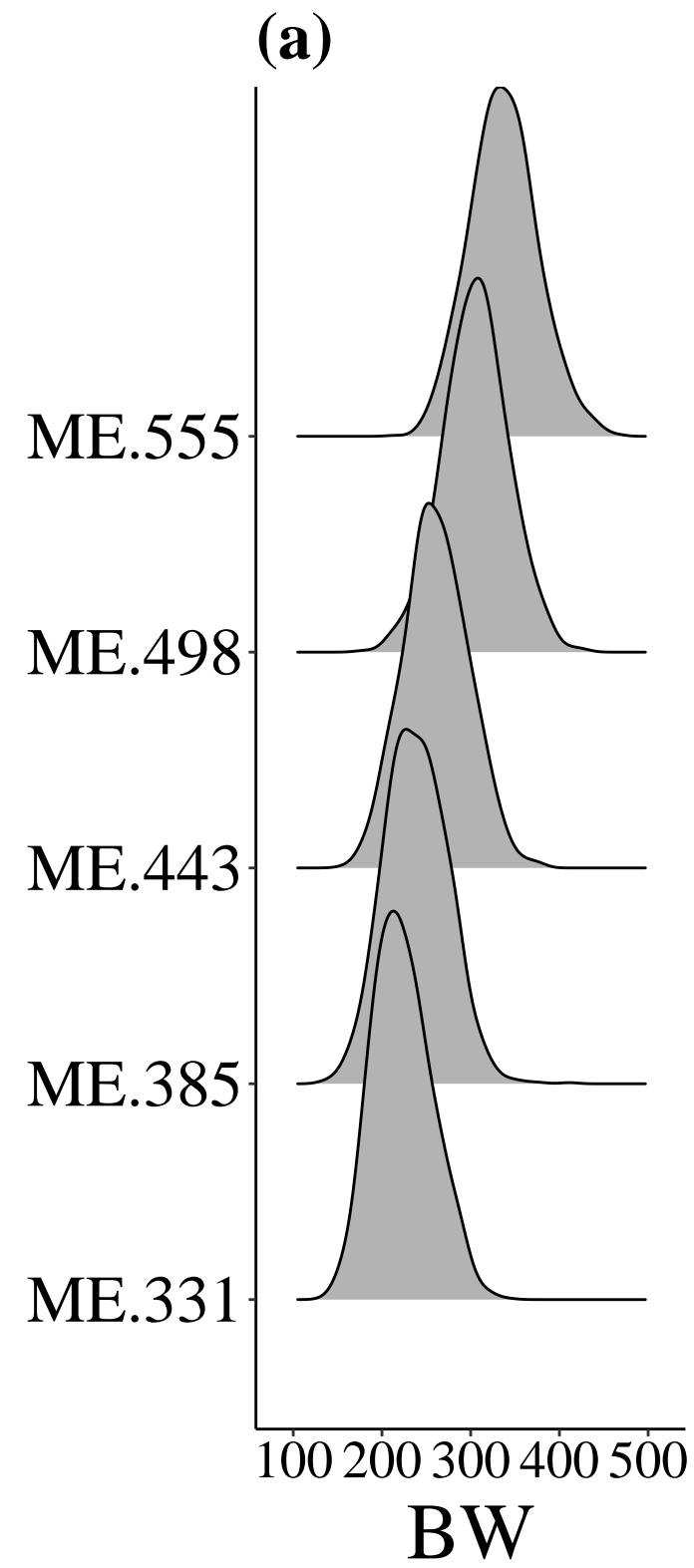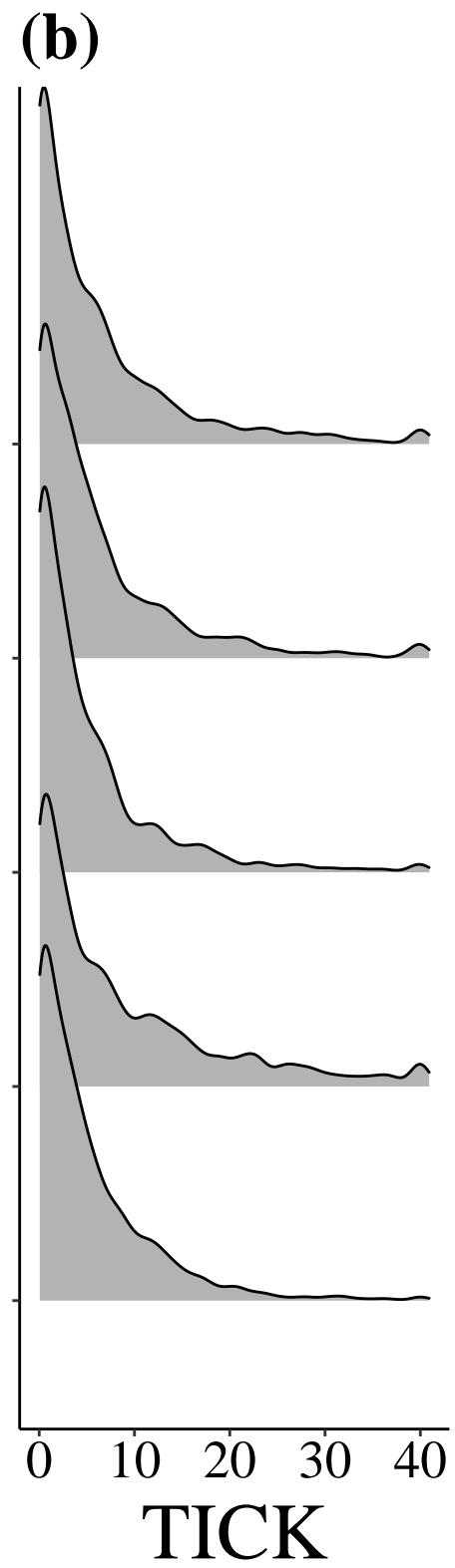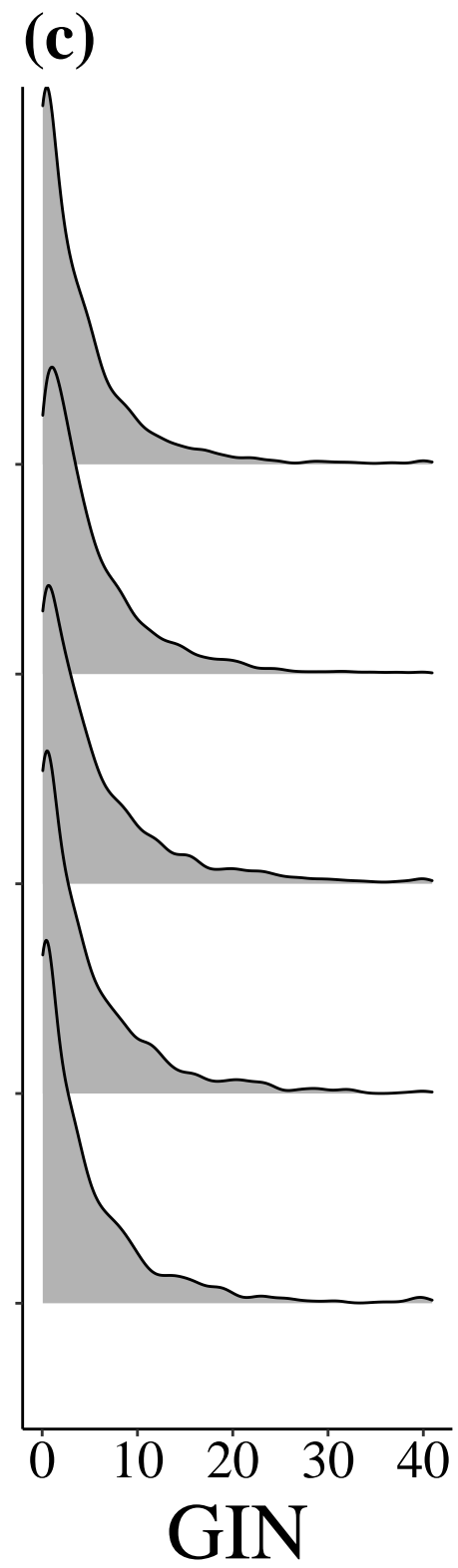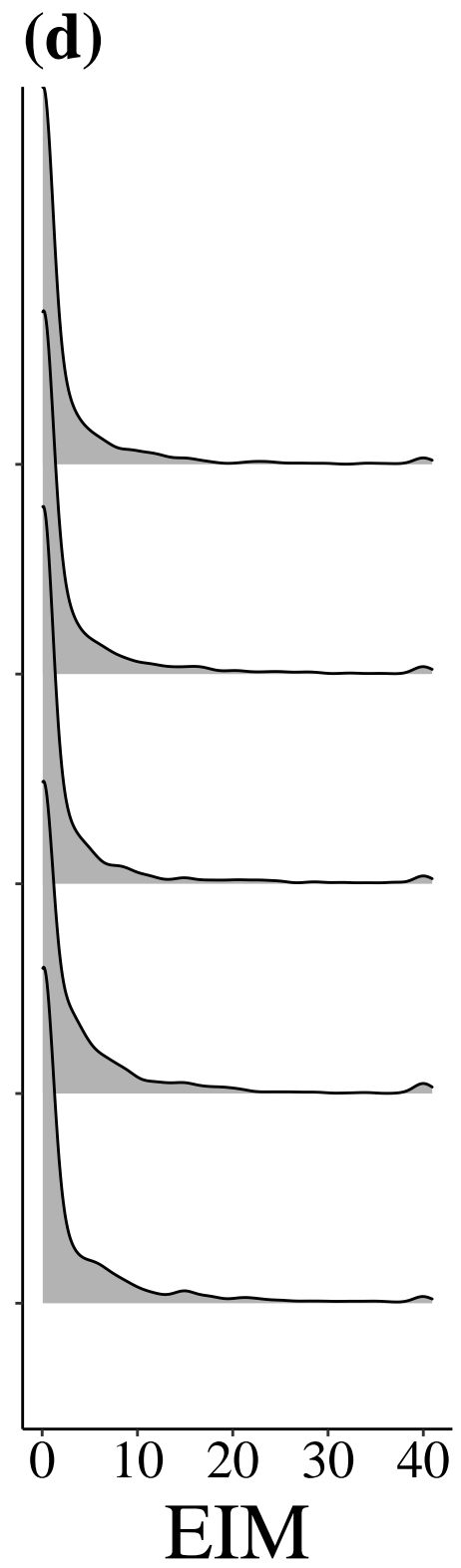

Supplement: Supplementary file 2 — Additional file 2: Figure S1. Distributions of body weight information (BW-a), ticks (TICK-b), gastrointestinal nematodes eggs (GIN-c), and Eimeria spp. oocysts (EIM-d) counts at each measurement event (ME). 331, 385, 443, 498, and 555 represent the mean ages of the animals at each ME, respectively. [file 12711_2023_844_MOESM2_ESM.pdf]

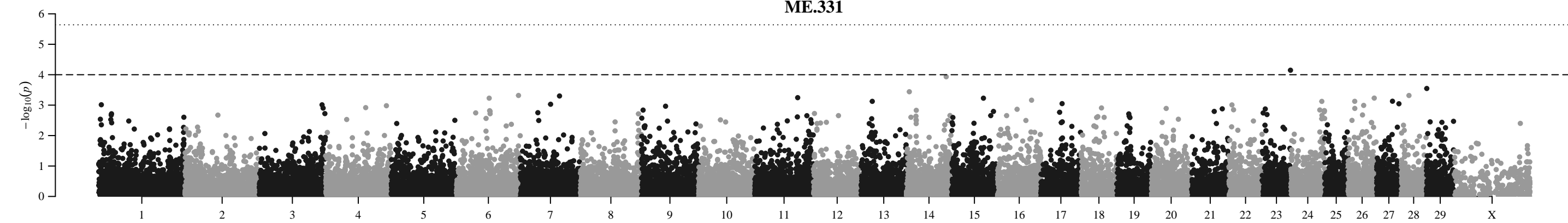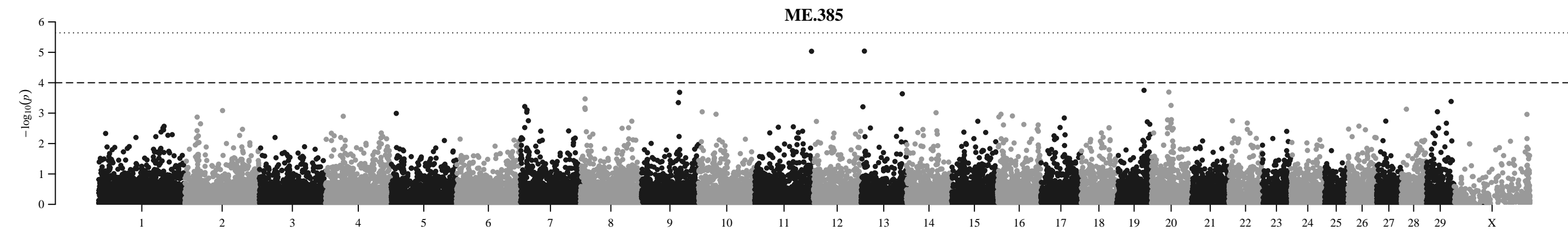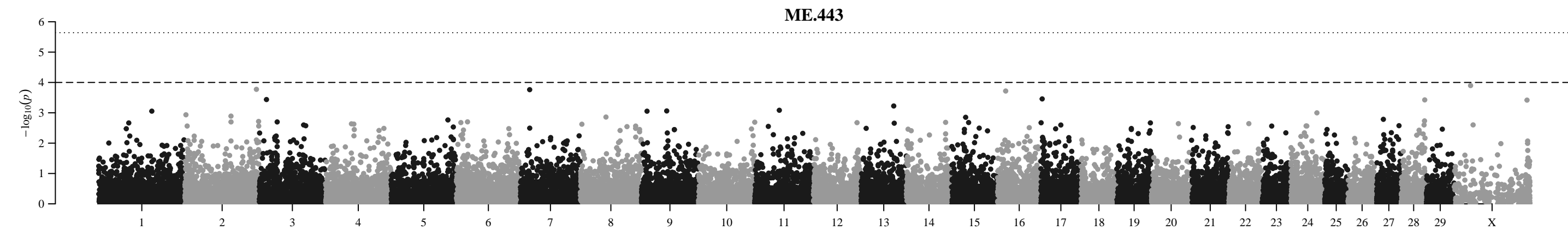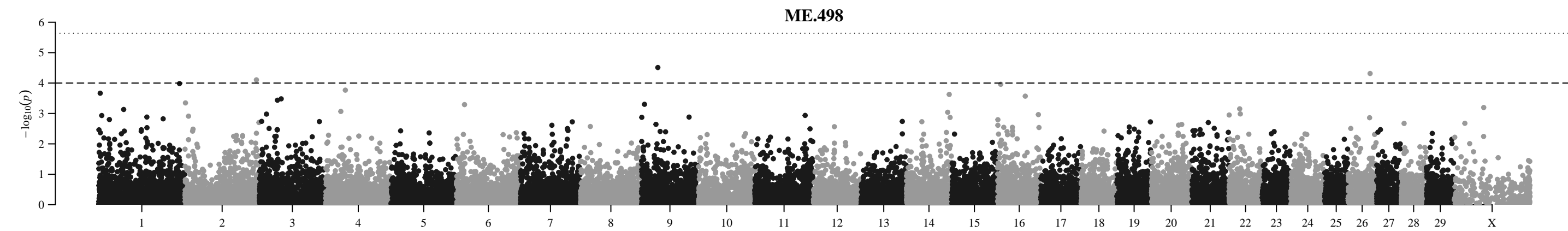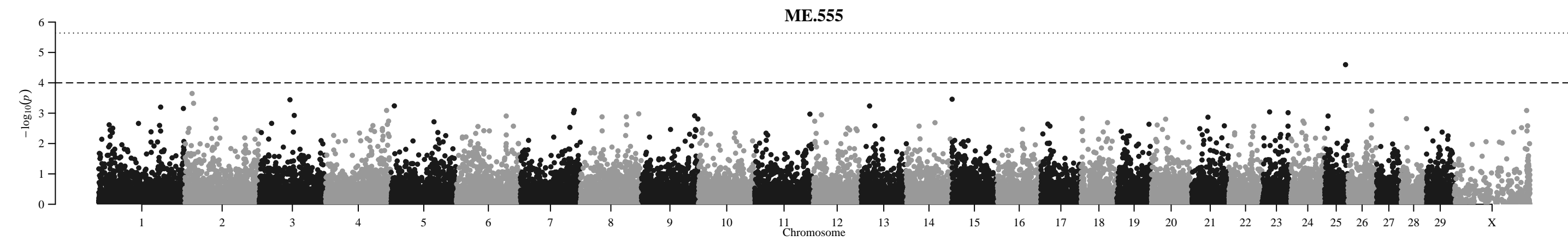

Supplement: Supplementary file 4 — Additional file 4: Figure S2. Manhattan plots for the genome-wide association studies for host tolerance to ticks evaluated at different measurement events (ME). 331, 385, 443, 498, and 555 are the mean ages (in days) of the animals at each ME. The dotted line (y = 5.64) indicates the threshold for statistical significance. The dashed line (y = 4.00) indicates the threshold for suggestive evidence of association. [file 12711_2023_844_MOESM4_ESM.pdf]

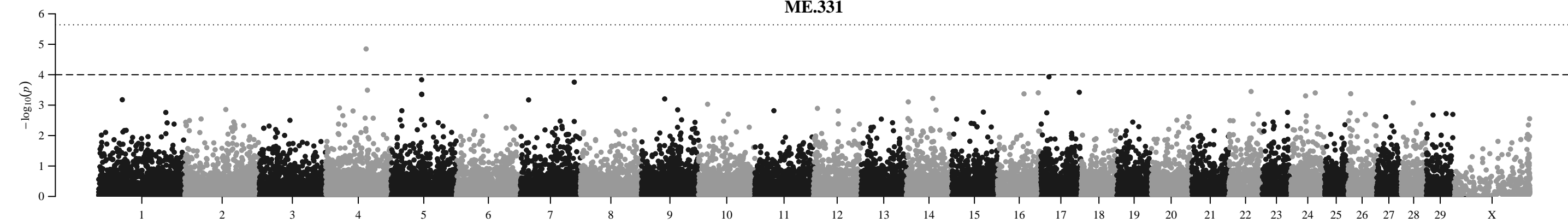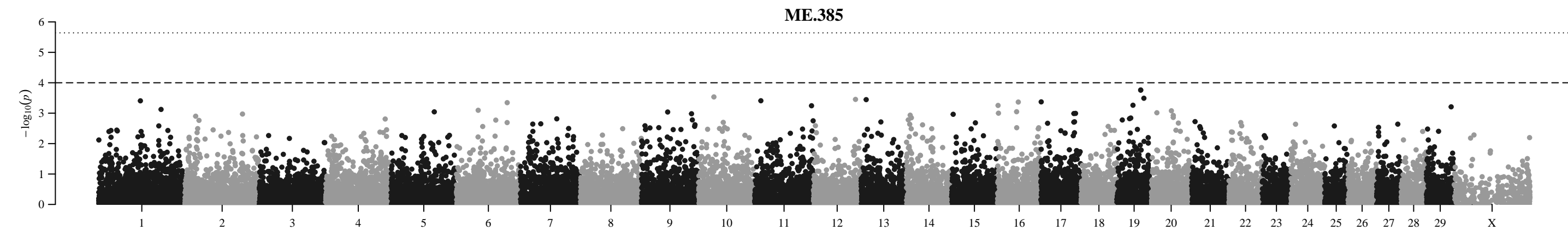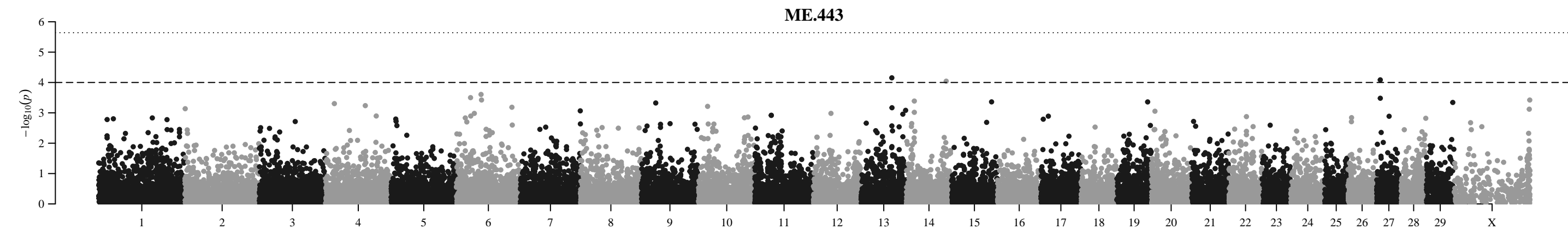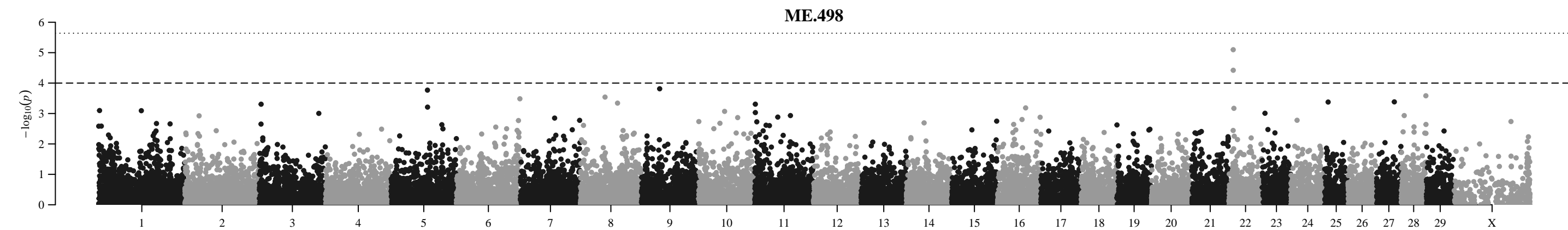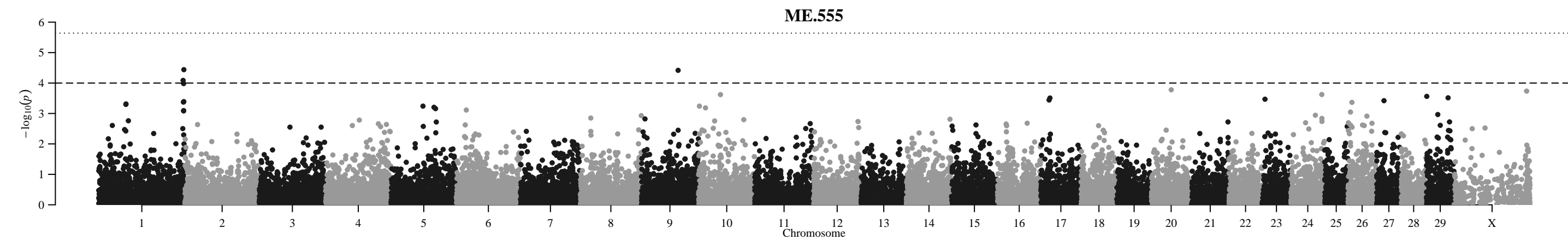

Supplement: Supplementary file 5 — Additional file 5: Figure S3. Manhattan plots for the genome-wide association studies for host tolerance to gastrointestinal nematodes evaluated at different measurement events (ME). 331, 385, 443, 498, and 555 are the mean ages (in days) of the animals at each ME. The dotted line (y = 5.64) indicates the threshold for statistical significance. The dashed line (y = 4.00) indicates the threshold for suggestive evidence of association. [file 12711_2023_844_MOESM5_ESM.pdf]

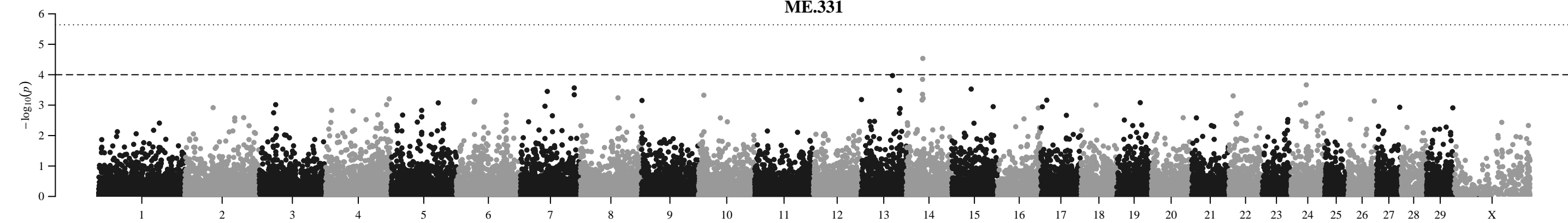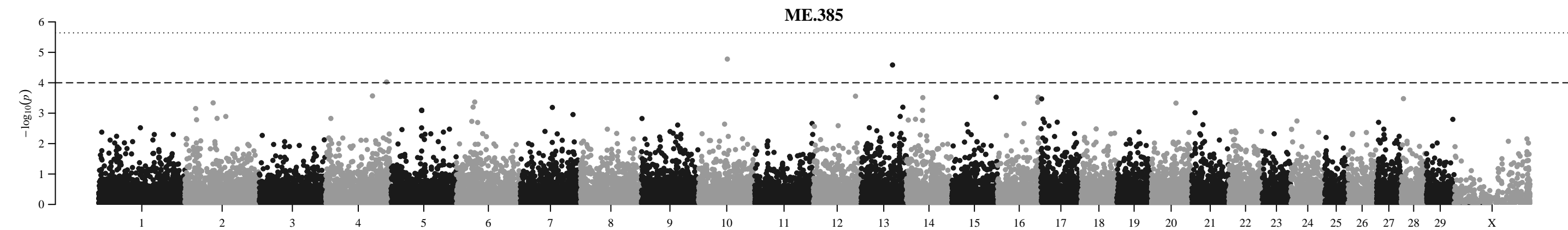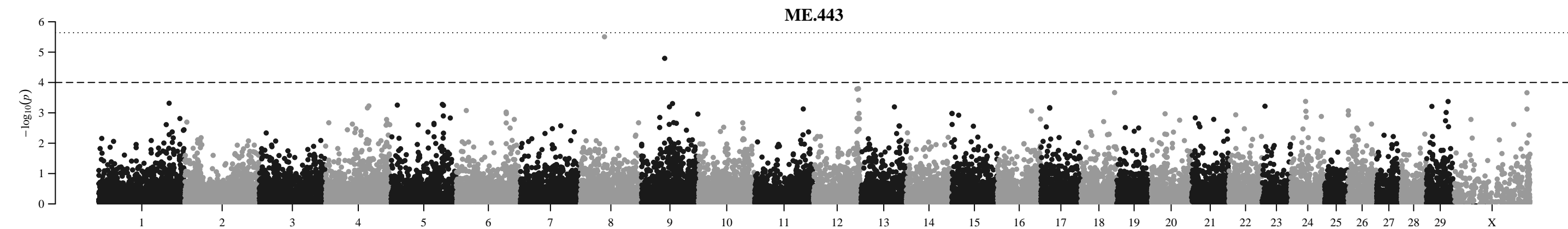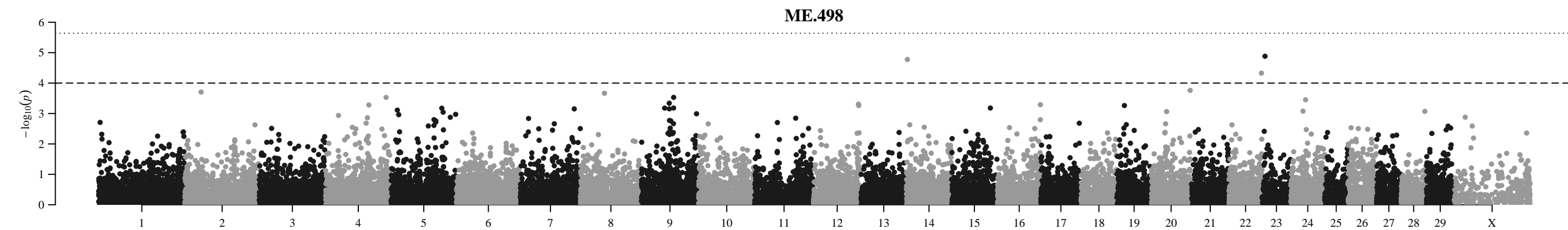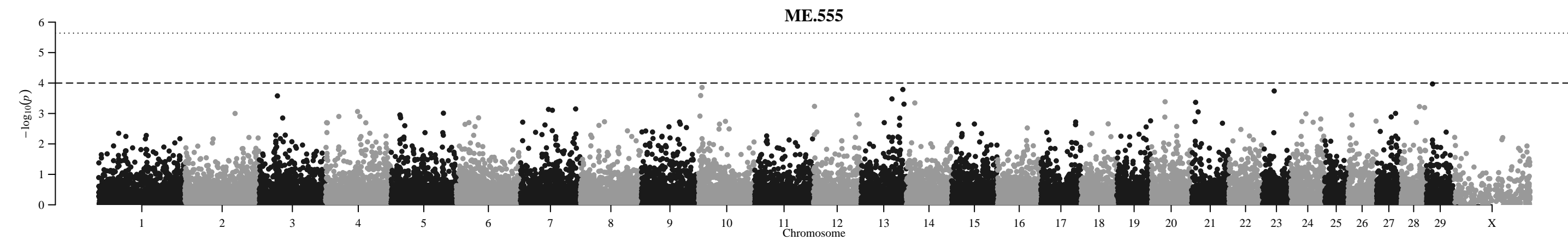

Supplement: Supplementary file 6 — Additional file 6: Figure S4. Manhattan plots for the genome-wide association studies for host tolerance to Eimeria spp. evaluated at different measurement events (ME). 331, 385, 443, 498, and 555 are the mean ages (in days) of the animals at each ME. The dotted line (y = 5.64) indicates the threshold for statistical significance. The dashed line (y = 4.00) indicates the threshold for suggestive evidence of association. [file 12711_2023_844_MOESM6_ESM.pdf]
