# Supplementary material for: Unravelling the genetic variability of host resilience to endo- and ectoparasites in Nellore commercial herds
Source: Genet Sel Evol. 2023 Nov 21;55:81. doi: 10.1186/s12711-023-00844-9 (PMC10664541; doi:10.1186/s12711-023-00844-9)
Supplement: Supplementary file 3 — Additional file 3. Additional information on the methods used to process the statistical analysis [60–65]. [file 12711_2023_844_MOESM3_ESM.docx]

**Additional file 3. Additional information on the methods used to process the statistical analysis**

We used single trait linear random regression models (STM) to estimate the genetic parameters for host resilience to different parasites and the genetic parameters for body weight across a parasite burden trajectory. The model was previously described in the Methods section of the main text, and the analysis was accomplished through Bayesian methods by using Gibbs sampler.

For the STM, the prior assumptions and distributions were:

*y* was assumed as $y|b,a,G0,R1\sim N(Xb+Za,IR1)$, where: *G0* is the covariance matrix for the genetic additive effects, which were previously described in the Methods section; R1 is the matrix of the residual effects, from which was considered homogeneity of variance so that $R1=\left[\sigma_{e_{1}}^{2}\right]$. *X* and *Z* are the incidence matrices for the systematic (*b*) and genetic additive effects (*a*), respectively; *N* is the normal distribution; and *I* is an identity matrix with order equal to the number of observations.

The prior distribution for the effects were: $b\sim constant$; $\left[ \begin{matrix} a_{0_{(i)}} & a_{1_{(i)}} \end{matrix} \right]^{t}|A,G0\sim N(\left[ \begin{matrix} 0 & 0 \end{matrix} \right]^{t},A\otimes G0)$; and $e|I,R1\sim N(0,R1)$. Inverted Wishart distribution was assumed for the covariance matrix G0 $(G0\sim IW(\Sigma_{a}^{2},n_{a})$ and Scaled inverse chi-squared distribution was assumed for $\sigma_{e}^{2}$ ($\sigma_{e}^{2}\sim\chi^{-2}(v_{e},S_{e}^{2})$, where A is the relationship matrix, $\Sigma_{a}^{2}$ and $n_{a}$ are the hyperparameters for the Inverted Wishart distribution and $v_{e}$ and $S_{e}^{2}$, are the hyperparameters for the scaled inverse chi-squared distribution. Non-informative priors were used. Information about a posteriori complete conditional distributions are also provided in Sorensen and Gianola [60].

Samples of the complete conditional distributions were obtained through the Gibbs sampler using the software GIBBS3F90 [61], with a chain of 1100000 iterations, discard of the 100000 first and sampling each 100 cycles. All the analyses were processed in sagarana HPC cluster, CEPAD-ICB-UFMG. The chain length was defined according to the method of Raftery and Lewis [62] in preliminary analysis, which is available in the BOA package [63] of the software R [26]. The convergence of the chains for each parameter of the model was evaluated by the criteria of Geweke [64] and Heidelberger and Welch [65], which are available in the same software and by visual inspection of the sampled values. For each parameter of the models the posterior means and high posterior density intervals with 90% of samples (HPD90) were calculated. The HPD90 was considered as a measure of uncertainty of the parameter estimate.

References

60. Sorensen D, Gianola D. Likelihood, Bayesian, and MCMC methods in quantitative genetics. New York: Springer-Verlag; 2002.

61. Misztal I, Tsuruta S, Lourenco D, Aguilar I, Legarra A, Vitezica Z. Manual for BLUPF90 family of programs. Athens: University of Georgia; 2015.

62. Raftery AE, Lewis SM. One long run with diagnostics: implementation strategies for Markov Chain Monte Carlo. Stat Sci. 1992;7:493–7.

63. Smith BJ. Boa: an R Package for MCMC output convergence assessment and posterior inference. J Stat Softw. 2007;21:1–37.

26. R Core Team. R: a language and environment for statistical computing. Vienna: R Foundation for Statistical Computing; 2019.

64. Geweke J. Evaluating the accuracy of sampling-based approaches to the calculation of posterior moments. Staff report 148. Minneapolis: Federal Reserve Bank of Minneapolis; 1991.

65. Heidelberger P, Welch PD. Simulation run length control in the presence of an initial transient. Oper Res. 1983;31:1109–44.
